# Effect of Different Basal Media and Organic Supplements on In Vitro Seedling Development of the Endangered Orchid Species *Dendrobium moniliforme* (L.) Swartz

**DOI:** 10.3390/plants13192721

**Published:** 2024-09-28

**Authors:** Jung Eun Hwang, Hyeong Bin Park, Dae Young Jeon, Hwan Joon Park, Seongjun Kim, Chang Woo Lee, Young-Joong Kim, Young-Jun Yoon

**Affiliations:** 1 Research Center for Endangered Species, National Institute of Ecology, Yeongyang 36531, Republic of Korea; phb1274@nie.re.kr (H.B.P.); uagin@nie.re.kr (D.Y.J.); rhg9281@nie.re.kr (H.J.P.); dao1229@nie.re.kr (S.K.); jacky903@nie.re.kr (C.W.L.); yjkim@nie.re.kr (Y.-J.K.); yjyoon@nie.re.kr (Y.-J.Y.); 2 Department of Environmental Horticulture, University of Seoul, Seoul 02504, Republic of Korea

**Keywords:** seedling growth, tissue culture, MS medium, hyponex medium, organic supplementation

## Abstract

The orchid *Dendrobium moniliforme* faces endangerment due to habitat loss and illegal harvesting, necessitating the development of an optimized artificial propagation system to aid conservation and reintroduction efforts. This study evaluated the effects of three plant growth media, namely Murashige and Skoog (MS), Hyponex, and Orchid Maintenance Medium (OMM) (P668), and various organic additives (apple homogenate, banana homogenate, and coconut water) on the in vitro seedling growth of *D. moniliforme*. The results reveal that, in early postgermination stages, seedlings achieve maximum growth in the Hyponex medium, with a fresh weight (92 mg) and root length (2.7 cm) approximately 20-fold greater than those in the MS medium and OMM. After 6 months, for seedlings grown in MS medium and OMM with banana (50 g·L^−1^), the mean fresh weights were 29 and 107 mg, respectively; however, the highest biomass was observed in seedlings grown in the Hyponex medium with coconut water (50 mL·L^−1^), exhibiting a mean fresh weight of 201 mg. This study highlights Hyponex medium with coconut water as the most effective combination for promoting *D. moniliforme* growth and identifies suitable organic supplements for the in vitro cultivation of seedlings from asymbiotic seed culture. This propagation system offers valuable technical support for the mass production and conservation of this epiphytic orchid.

## 1. Introduction

The *Orchidaceae* family is one of the largest and most widespread among angiosperms, with 29,524 recognized species, representing 8% of angiosperm diversity [1,2,3]. However, approximately 25% of all orchid species face extinction due to habitat loss, smuggling, climate change, and overexploitation, making orchids the most threatened plant group globally [4]. Therefore, their conservation has become a global concern. Natural orchid propagation is limited by low seed germination and slow growth; hence, in vitro tissue culture is considered an effective conservation method [5,6].

Orchid asymbiotic seed germination is markedly influenced by several factors, including embryo developmental stage, pod maturity, and the use of nutrient media containing additives and plant growth regulators [7]. Adding specific plant growth regulators, activated carbon, and peptone, along with adjusting culture medium composition, enhances germination and protocorm development in many orchids [8]. Organic growth supplements also impact the in vitro regeneration, multiplication, and growth of orchid seedlings [9]. Organic additives, including apple juice, banana homogenate, potato homogenate, coconut water, tomato juice, honey, corn extract, yeast extract, and papaya extract, have effectively enhanced the development of cultured cells and tissues in prior studies [10,11]. In orchid production, numerous organic amendments have proven successful [12,13,14,15]. Given that these components vary by species, their concentrations in the medium must be tailored to each orchid species’ specific requirements [16,17,18]. Therefore, this study aimed to identify the most effective additives for promoting the in vitro growth of *Dendrobium moniliforme* (L.) Swartz seedlings during asymbiotic seed culture.

**Figure 1:**
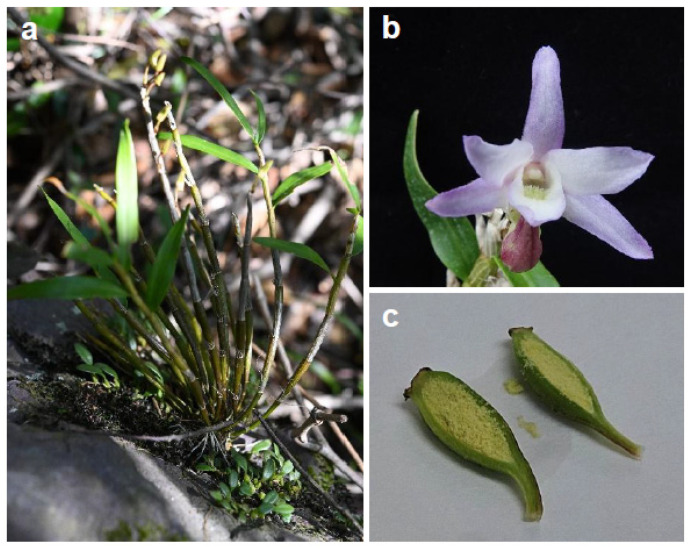
(**a**) *Dendrobium moniliforme* plant. (**b**) Flower. (**c**) Immature seed capsule.

*Dendrobium moniliforme* is a perennial epiphyte and one of the most widely distributed species within the *Dendrobium* genus. It is predominantly found in East Asia, including China, Japan, and Korea [19,20]. As a typical epiphyte, this orchid requires minimal amounts of water and nutrients. Its flowers bloom around May–June, usually being white or light pink [21] (Figure 1b). Additionally, *D. moniliforme* has demonstrated antioxidant [22] and neuroprotective [23] effects, with other medicinal properties under investigation. Its dual role as both an ornamental and medicinal plant underscore the economic importance of *D. moniliforme* [21].

Like many orchids, *D. moniliforme* is threatened by overexploitation, habitat loss, and deforestation. In Korea, the species is particularly endangered, leading to its designation as a Class 2 endangered wild species by the Ministry of Environment, granting it legal protection. In response, several studies have focused on the mass propagation of *D. moniliforme* using plant tissue culture techniques. Previous research has explored suitable basal media for seed germination, the impact of varying sucrose concentrations, and the effects of organic additives on growth of plants [24]. Additionally, activated charcoal and sucrose have been shown to markedly enhance shoot proliferation and rooting in *D. moniliforme* [25]. Other studies have investigated plant growth regulators in promoting protocorm-like body (PLB) formation and overall growth [25,26]. Despite these advances, the specific effects of various organic supplements on *D. moniliforme* growth remain underexplored.

This study examines the impact of different basal media and organic supplements on seedling growth, aiming to establish a reproducible protocol for rapid and efficient *D. moniliforme* propagation through in vitro culture. The findings are expected to contribute to conservation efforts by providing a reliable method for generating plantlets suitable for reintroduction into natural habitats. By optimizing organic supplements in the culture medium, this study contributes to enhancing orchid propagation efficiency, ultimately supporting the preservation and restoration of this endangered species.

## 2. Results and Discussion

### 2.1. Effects of Various Basal Media on Seedling Growth

The in vitro growth of orchid seedlings is heavily influenced by the composition of the culture medium employed. Several basal media formulations, including Knudson C [27], Vacin and Went [28], Hyponex [29], Orchid Maintenance Medium [30], and Murashige and Skoog [31] medium, have historically been used for cultivating various orchid species. These media supply essential nutrients for orchid seed germination and seedling development. Previous studies, including our own, have shown that MS medium, Hyponex medium, and Orchid Maintenance Medium (OMM) are particularly effective for promoting orchid seedling growth [15,32]. In the current study, we evaluated those media to identify the most suitable one for the in vitro growth of *D. moniliforme*.

**Figure 2:**
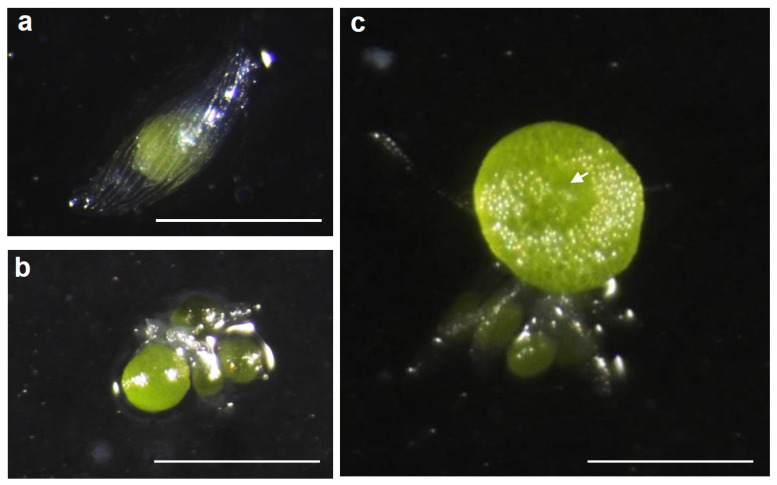
*Dendrobium moniliforme* seed germination and protocorm development. (**a**) Swollen seed embryos. (**b**) Ruptured seed coat. (**c**) Shoot apices (indicated by arrows) formed from embryos. Scale bar = 500 μm.

Initially, *D. moniliforme* seeds were germinated on Hyponex medium. According to the results, the seeds of *D. moniliforme* exhibited a high germination rate of over 98%. These findings suggest that *D. moniliforme* seeds possess a strong ability to germinate. The protocorm and shoot apex developed after 3 weeks (Figure 2b) and 4 weeks (Figure 2c) after sowing, respectively. In our study, the developmental stages of the protocorm progressed similarly to those described by Liu et al. [33].

**Figure 3:**
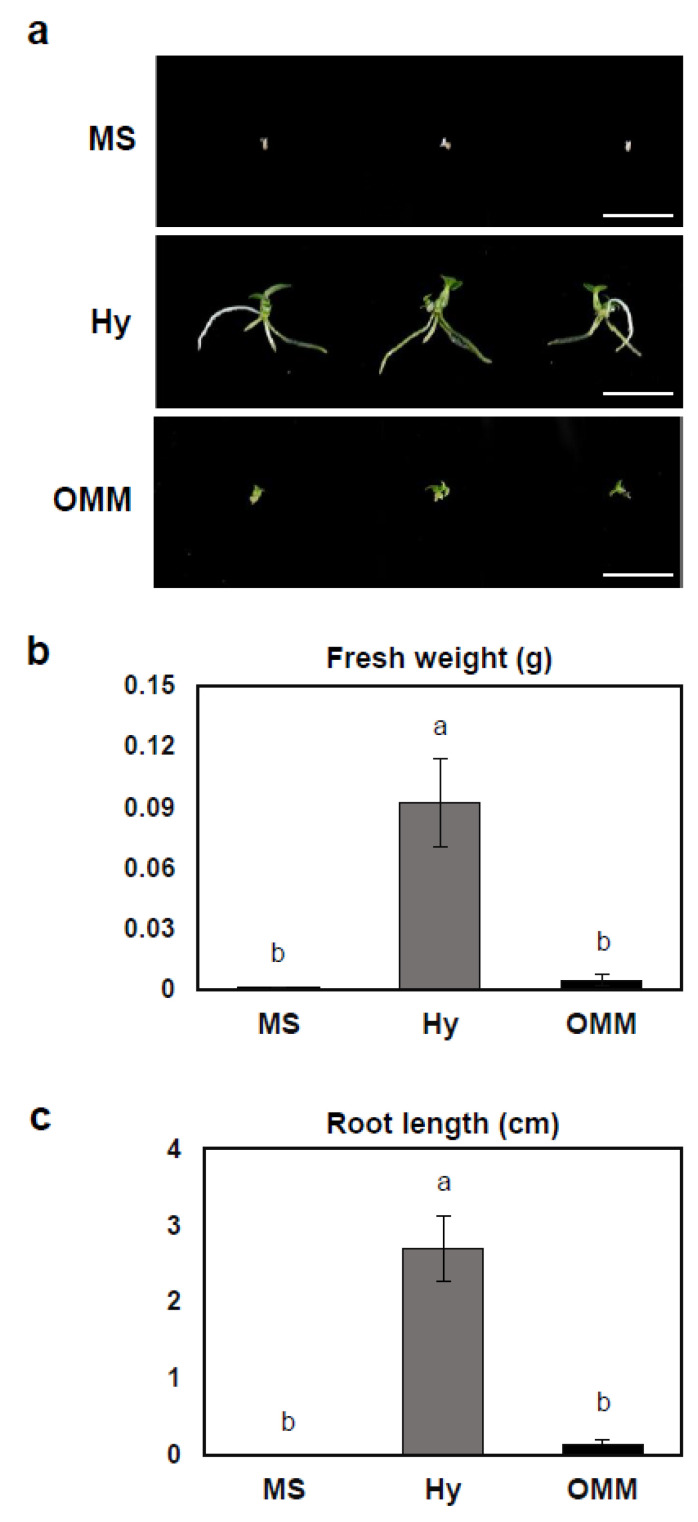
Effects of culture media on *D. moniliforme* seedling growth. (**a**) Growth differences in seedlings were assessed 5 months postsowing. Images show a representative seedling from each medium. (**b**) Fresh weight of plantlets and (**c**) root length. Scale bar: 2 cm. MS: Murashige and Skoog; Hy: Hyponex; OMM: Orchid Maintenance Medium. Data are presented as means ± standard deviations (SDs) from triplicate experiments. Letters indicate the significant differences based on Tukey’s Honestly Significant Difference (HSD) test (*p*-value < 0.05).

Two months after sowing, protocorms at the same developmental stage were transferred to MS medium, Hyponex medium, and OMM. As illustrated in Figure 3a, plants grown on MS and OMM showed minimal growth, with limited shoot and root development. In contrast, plants grown on Hyponex medium exhibited the most vigorous growth, with well-formed shoots and significantly longer roots, indicating that Hyponex provides a more favorable environment for plant development.

To evaluate overall biomass accumulation, the fresh weight was measured (Figure 3b). Seedlings grown on Hyponex medium had substantially a higher fresh weight value compared to those grown on MS and OMM, with fresh weight in Hyponex being nearly 20-times greater. Likewise, the root length was significantly longer in plants grown on Hyponex, with an average length of approximately 3 cm, whereas the root length in MS and OMM remained minimal, both close to zero (Figure 3c).

These findings suggest that the choice of growth medium has a substantial effect on seedling development. Among the media tested, Hyponex consistently supported the best growth, both in terms of fresh weight and root length, indicating it provides more suitable conditions for plant development compared to MS and OMM.

Several studies have investigated the germination and growth of *D. moniliforme*. Liu et al. [26] and Bae et al. [25] used MS medium for *D. moniliforme* germination and plantlet production. However, these studies did not compare different basal media, focusing instead on optimizing MS medium for specific growth parameters. Bae et al. [25] additionally examined the effects of naphthaleneacetic acid and indole-3-butyric acid concentrations on PLB (protocorm-like body) induction in seedlings cultured on MS medium for eight weeks. They found that MS medium with those regulators effectively promoted PLB formation, whereas MS medium lacking them did not induce such formation. This suggests that, although MS medium can support *D. moniliforme* growth under certain conditions, it may not be as effective as Hyponex medium for overall seedling development.

Hyponex is a commercially available fertilizer commonly used in orchid seed germination and plant regeneration media. It has been shown to enhance various growth parameters in orchids. For instance, Dwiyani [34] reported that Hyponex fertilizer increased plant height and leaf number in *Dendrobium* species, and Thepsithar et al. [35] found that it improved the number of seedlings. Conversely, Alam et al. [36] noted that MS medium outperformed Hyponex for specific *Dendrobium transparens* growth characteristics. However, our study, alongside the findings from Nitsch et al. [37], who reported that Hyponex medium was suitable for plant regeneration in the PLB of *Phalaenopsis*, suggests that Hyponex effectively promotes *D. moniliforme* plant height and root number.

Seedling growth differences observed across various media can be attributed to the media’s distinct compositions. Although MS medium, Hyponex medium, and OMM all contain similar fundamental components, including carbohydrates and mineral salts, they differ in terms of specific types and concentrations of mineral salts, organic additives, and vitamins. MS medium and OMM are enriched with macronutrients and micronutrients, essential for plant growth. However, in the present study, the richness of these nutrients in the media may have inhibited effective *D. moniliforme* growth, possibly due to nutrient imbalance or osmotic stress.

The form and availability of nitrogen in the culture medium are critical factors influencing orchid seedling growth. Nitrogen is vital for plant growth, contributing to protein synthesis, enzyme activity, and chlorophyll production. The nitrogen source, whether organic or inorganic, markedly affects orchid germination and growth. Previous studies have shown that orchids employ diverse nitrogen metabolic pathways, with some species thriving on inorganic nitrogen sources, such as ammonium and nitrate, whereas others perform better with organic nitrogen sources [38,39,40].

In our study, MS and Hyponex media contained only inorganic nitrogen, i.e., ammonium and nitrate or potassium nitrate, whereas OMM included a mixture of organic and inorganic nitrogen. The results reveal that *D. moniliforme* germination and primary shoot development are more effectively promoted by Hyponex medium, suggesting that this species efficiently utilizes the inorganic nitrogen provided by this medium. This supports the hypothesis that *D. moniliforme* may favor inorganic nitrogen sources, which are readily available and easily absorbed.

In conclusion, our results demonstrate that Hyponex is the most suitable basal medium for the in vitro growth of *D. moniliforme* seedlings. Its simplicity, combined with its provision of essential nutrients, especially inorganic nitrogen, makes it conducive to promoting *D. moniliforme* growth. These findings have implications for the conservation and propagation of this endangered species, offering a reliable method for producing healthy plants suitable for reintroduction into natural habitats.

### 2.2. Effects of Organic Supplements on Seedling Growth

Organic supplements are well-known for enhancing plant tissue growth in vitro, particularly in orchid culture. These supplements, including various fruit homogenates and plant extracts, are added to culture media as natural carbon sources, as well as for their vitamins, fibers, hormones, and proteins [10]. Previous studies have highlighted the effectiveness of organic additives, such as apple homogenate, banana homogenate, potato homogenate, coconut water, papaya extract, and corn extract, in promoting orchid development [10,11,41,42]. In this study, we evaluated the effects of apple homogenate, banana homogenate, and coconut water on *D. moniliforme* seedling growth.

Seedling growth was monitored at 4, 5, and 6 months after sowing (Table 1). The study investigated the effects of organic supplements—apple homogenate (AH), banana homogenate (BH), coconut water (CW), and a mixture of these three (ABC)—on the seedling growth of *Dendrobium moniliforme*. As shown in Table 1, the fresh weight of seedlings grown in MS and OMM remain relatively low at 4, 5, and 6 months, even with the addition of organic supplements. The highest fresh weight observed on MS medium after 6 months was 29 mg, with the addition of BH, while the highest fresh weight on OMM was 107 mg with BH. These values were improvements over the untreated control, but were still considerably lower than those observed on Hyponex medium. In contrast, when CW was added to the Hyponex medium, the fresh weight reached 201 mg after 6 months, the highest among all treatments (Table 1).

Root length followed a similar pattern in MS and OMM. The longest root length observed on MS medium after 6 months was 1.08 cm with BH, whereas the longest root length on OMM was 3.1 cm, also with BH (Table 1). Fresh weight was most enhanced in the Hyponex and CW combination medium, while root development was the best in the same medium and the control (4.0 and 3.12, respectively), after 6 months, though no significant difference was observed. Similar results were found at 4 and 5 months (Table 1). These results suggest that the Hyponex basal medium may provide sufficient nutrients for root development, as significantly longer root lengths were observed in Hyponex compared to MS and OMM.

**Figure 4:**
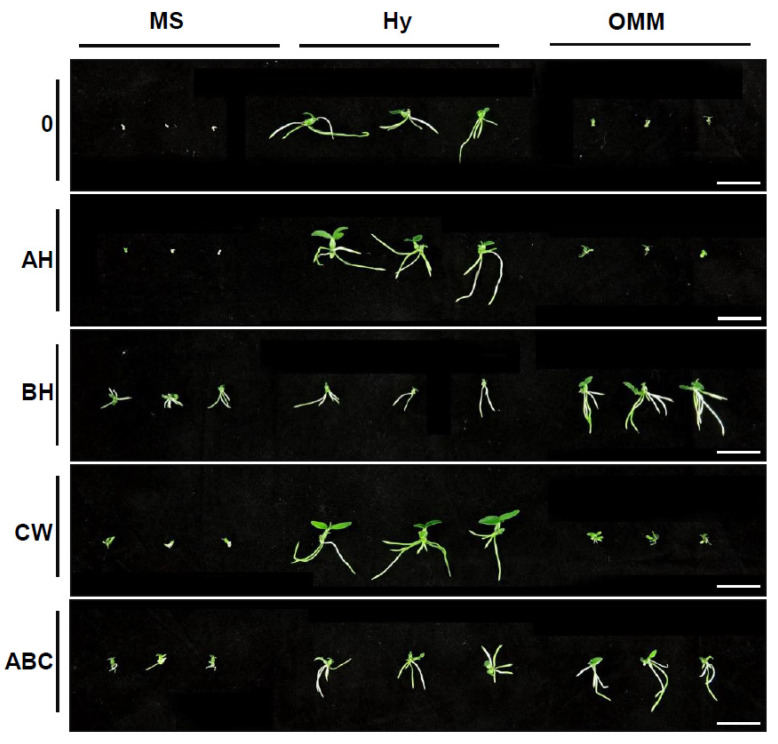
Effect of organic supplements on *D. moniliforme* seedling growth. Growth differences in seedlings determined 6 months postsowing. Images depict a representative seedling from each medium. Scale bar: 2 cm. MS: Murashige and Skoog; Hy: Hyponex; OMM: Orchid Maintenance Medium. AH: apple homogenate; BH: banana homogenate; CW: coconut water; ABC: all three organic supplements.

These findings emphasize that the effect of organic supplements on seedling growth are dependent on the basal medium used. While BH produced the highest growth rates in MS and OMM, CW had the most pronounced impact when combined with the Hyponex medium (Table 1 and Figure 4). This variability underscores the importance of selecting the appropriate combinations of media and organic supplements to optimize the growth of specific orchid species under in vitro culture conditions.

Coconut water and banana powder are well-known organic additives used in plant tissue culture due to their rich nutrient profiles, which provide a wide range of beneficial compounds that promote plant growth. Coconut water has been used as an organic additive in plant tissue culture since the early 1940s. Its role in enhancing the growth of various orchids, including *Phalaenopsis amabilis*, is well-documented [43]. Similar findings were reported by Winarto and da Silva [43] and Baque et al. [44], further validating coconut water’s efficacy in orchid culture.

Coconut water is a natural liquid endosperm that contains an array of beneficial compounds, including cytokinins, auxins, and gibberellins, which are critical plant hormones involved in cell division, root formation, and overall growth regulation [45]. Some of the most important and useful components of coconut water in plant tissue culture are cytokinins, a type of plant hormone [46]. Cytokinins are plant hormones that play an important role in plant growth and development, including cell division, the formation and activity of shoot meristems, the induction of photosynthetic gene expression, leaf senescence, nutrient transport, and seed germination. It has been reported that cytokinins play an essential role in plant morphogenesis by regulating root and shoot growth in combination with auxin [45]. However, cytokinins also have a negative regulatory function on root growth by inhibiting cell division in roots, unlike their role in promoting shoot growth [45]. This was evident in our study, where coconut water promoted shoot growth (Figure 4), but was less effective in root growth (Table 1).

Banana homogenate is frequently used as an organic additive in in vitro cultures due to its high content of potassium, manganese, calcium, sodium, iron, zinc, thiamine, riboflavin, niacin, pyridoxine, pantothenic acid, ascorbic acid, folic acid, and natural growth regulators, such as zeatin, gibberellin, and IAA. It is also rich in carbohydrates, which provide energy to heterotrophic plants in the early stages of in vitro cultivation [47]. Vyas et al. [48] and Arditti [49] et al. reported that the plant growth-promoting effect of bananas is due to these plant hormones. Tawaro [50] noted that iron in bananas exists in a usable form that stimulates growth and root formation in orchids. Despite some components of banana homogenate being transformed under high-temperature and -pressure conditions, thermostable substances remain active and promote plant growth [51]. While there are differing opinions on which substances are most effective, there is a consensus that banana homogenate promotes plant growth, aligning with our study’s findings for *D. moniliforme* on MS and OMM. These results are consistent with reports of significant plant growth promotion in *Renanthera imschootiana*, *Paphiopedilum wardii*, and *Phalaenopsis amboinensis* [52,53,54]. Thus, banana homogenate, like coconut water, is a valuable additive for supporting the in vitro production of *D. moniliforme.*

This study underscores the critical role played by organic supplements in optimizing growth conditions for *D. moniliforme* seedlings. The substantial increases in seedling fresh weight achieved with coconut water and banana homogenate suggest that these additives can contribute to enhancing in vitro orchid propagation. Given the endangered status of *D. moniliforme*, the application of such optimized growth media could be crucial in conservation efforts, enabling the mass propagation of healthy seedlings for reintroduction into natural habitats. In conclusion, our research demonstrates that coconut water and banana homogenate, in an optimal combination with basal medium, are effective for promoting the growth of *D. moniliforme* seedlings in vitro, providing valuable insights for improving culture protocols, benefiting the conservation and commercial propagation of this, and potentially other, endangered orchid species.

### 2.3. Effects of Various Media on Plant Growth at Later Development Stages

**Figure 5:**
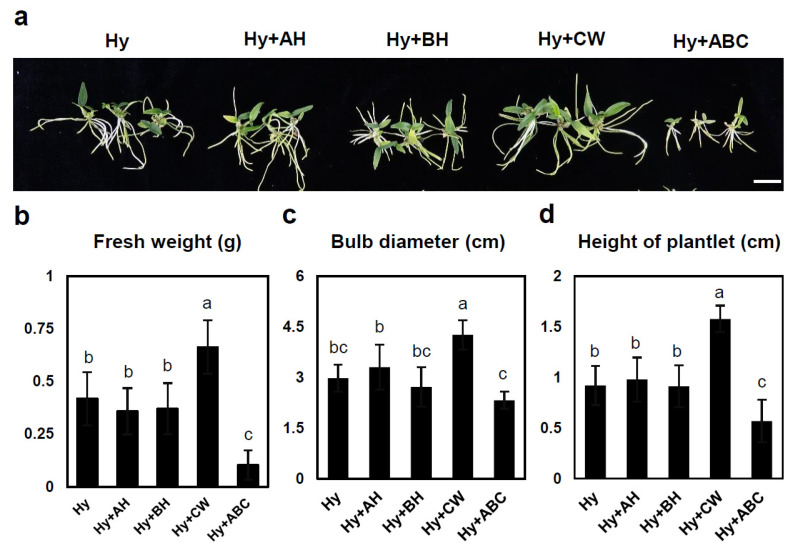
Effects of various media on plant growth at later stages of *D. moniliforme* development. (**a**) Growth differences in plantlets assessed 8 months postsowing. Fresh weight (**b**), bulb diameter (**c**), and plantlet height (**d**). Scale bar: 2 cm. MS: Murashige and Skoog; Hy: Hyponex; OMM: Orchid Maintenance Medium; AH: apple homogenate; BH: banana homogenate; CW: coconut water; ABC: all three organic supplements. Data are presented as means ± SDs from triplicate experiments. Letters indicate the significant differences based on Tukey’s Honestly Significant Difference (HSD) test (*p*-value < 0.05).

The influence of various culture media on *D. moniliforme* seedling growth was further investigated 8 months postsowing. This stage is crucial for assessing the long-term effects of media and organic supplements on plant vigor and health. An earlier experiment identified Hyponex as the optimal medium for early seedling growth, especially when supplemented with coconut water. To determine if these benefits persisted into later stages, we continued the experiment using the same medium. The results show that adding coconut water to Hyponex medium not only improves early growth, but also positively affects later developmental stages. Specifically, the fresh weight of plantlets in the Hyponex medium with coconut water increased 1.6-fold compared with those in Hyponex medium lacking the supplement. Similarly, the bulb diameter and plant height increased 1.4- and 1.7-fold, respectively, in the coconut water-supplemented medium (Figure 5). Thus, the benefits of coconut water observed during early growth stages were sustained and even amplified as the plants matured.

The observed increases in fresh weight, bulb diameter, and plant height indicate that coconut water in Hyponex medium supports not only initial seedling establishment, but also further development into robust plants. This sustained growth may be attributed to coconut water’s nutritional profile, where growth-promoting hormones, including cytokinins, are present. Growth was specifically promoted in the shoot portion of the plant. This is thought to be due to the role of cytokinins in promoting growth in the shoot apical tissue of the plant, as mentioned above [45].

This study provides valuable insights into the role of organic supplements in orchid cultivation, particularly for species similar to the study subject, *D. moniliforme*, being both ornamental and endangered. Our results demonstrate that selecting the appropriate medium and organic supplement is crucial not only for initial germination and seedling growth, but also for the sustained development of plants. The continued positive impact of coconut water on growth parameters at later stages underscores its potential as a key component in orchid tissue culture protocols for conservation and mass propagation.

## 3. Materials and Methods

### 3.1. Plant Material and Surface Sterilization of Capsules

*Dendrobium moniliforme* seeds were collected from plants growing in early August from Hongdo Island, Korea (Figure 1a). Initially, the collected immature green seed capsules were washed with a mild detergent solution to remove surface impurities. They were then rinsed three times with running tap water to ensure complete detergent removal. The capsules were subsequently transferred to a clean bench for surface sterilization. They were immersed in 70% ethanol for 3 min, rinsed 3 times with sterile distilled water to remove residual ethanol, immersed in 12% sodium hypochlorite (NaOCl) solution for 15 min with continuous agitation, and rinsed 5 times with sterile distilled water to eliminate residual NaOCl. Under aseptic conditions, the sterilized capsules were cut longitudinally using a sterile scalpel blade (Figure 1c). Seeds were carefully extracted and placed into a sterile 50 mL conical tube. To prepare the seed suspension, 30 mL of sterile distilled water was added, and the tube was gently agitated to evenly distribute the seeds in the suspension. Using a sterilized pipette, 200 μL of the seed suspension was transferred as a single drop onto the surface of 100 mL of culture medium in a deep, square culture bottle (SPL, Seongnam, Republic of Korea). This process was performed under strict aseptic conditions to avoid contamination. Germination was carried out on Hyponex medium in a growth chamber maintained at 24 °C ± 2 °C with a 16/8 h light/dark cycle using fluorescent lamps (120 μE s^−1^ m^−2^).

### 3.2. Culture Media for In Vitro Seedling Growth

To evaluate the effects of various basal media on seedling growth, 15 protocorms at the same developmental stage (2 months postsowing) were cultured in 3 media: (1) MS with vitamins (Duchefa, Haarlem, The Netherlands), (2) Hyponex (N:P:K = 7:6:19; 3 g·L^−1^ (Kisan Bio, Seoul, Republic of Korea), and (3) OMM (Orchid Maintenance Medium; PhytoTechnology, Lenexa, KS, USA; P668).

To evaluate the impact of organic supplements on seedling growth, protocorms at the same developmental stage (2 months postsowing) were cultured on the 3 basal media supplemented with apple homogenate (Kisan Bio, Seoul, Republic of Korea) at 10 g·L^−1^ (AH), banana homogenate (Kisan Bio, Seoul, Republic of Korea) at 30 g·L^−1^ (BH), and coconut water (Kisan Bio, Seoul, Republic of Korea) at 50 mL·L^−1^ (CW), as well as a combination of all 3 organic supplements (ABC). The media compositions were as follows: (1) MS with AH 10 g·L^−1^; (2) MS with BH 30 g·L^−1^; (3) MS with CW 50 mL·L^−1^; (4) MS with AH 10 g·L^−1^, BH 30 g·L^−1^, and CW 50 mL·L^−1^; (5) Hyponex with AH 10 g·L^−1^; (6) Hyponex with BH 30 g·L^−1^; (7) Hyponex with CW 50 mL·L^−1^; (8) Hyponex with AH 10 g·L^−1^, BH 30 g·L^−1^, and CW 50 mL·L^−1^; (9) OMM with AH 10 g·L^−1^; (10) OMM with BH 30 g·L^−1^; (11) OMM with CW 50 mL·L^−1^; and (12) OMM with AH 10 g·L^−1^, BH 30 g·L^−1^, and CW 50 mL·L^−1^. Media without organic supplementation were used as controls. All media were supplemented with 20 g·L^−1^ sucrose and 1% activated charcoal and solidified with 0.8% (*w*/*v*) agar. The pH was adjusted to 5.6, and the media were autoclaved at 120 °C for 15 min.

Cultures were maintained in a growth chamber at 24 °C ± 2 °C under a photoperiod of a 16/8 h light/dark cycle with fluorescent lamps (120 μEs^−1^m^−2^). Subcultures were transferred to fresh media every two months. Fresh weight of the whole plant and root length were recorded regularly. The entire experiment was performed under sterile conditions under a laminar airflow hood to prevent contamination.

### 3.3. Measurement of Seedling Development and Statistical Analysis

Each treatment consisted of three independent replicates, with five seedlings per replicate. After 4, 5, 6, and 8 months, seedling fresh weight, root length, and root number were recorded. Data were analyzed using one-way and two-way analyses of variance with a post hoc Tukey’s Honestly Significant Difference test, with the results considered statistically significant at *p* ≤ 0.05. Statistical analyses were performed using SAS 9.4 statistical software (SAS Institute Inc., Cary, NC, USA).

## 4. Conclusions

This study demonstrates that asymbiotic culture techniques can significantly enhance the in vitro growth and development of *D. moniliforme* seedlings. Our findings highlight the importance of both basal medium selection and organic supplement addition across various stages of plant development. Among the media tested, Hyponex medium supplemented with coconut water showed the best results for *D. moniliforme* growth. These findings are particularly relevant for the conservation and restoration of *D. moniliforme*, given its endangered status. By optimizing culture conditions using effective organic additives, such as coconut water, we can improve seedling growth. Moreover, these insights can be applied to other orchid species, enhancing broader conservation efforts within the Orchidaceae family.

In conclusion, this study provides a detailed understanding of the factors influencing *D. moniliforme* development in vitro. The use of Hyponex medium with coconut water represents an advancement in orchid propagation techniques. This approach will be valuable for researchers and conservationists focused on preserving endangered orchid species, offering a practical method to enhance plant growth and ensure their long-term survival.

## Figures and Tables

**Table 1:** Effects of organic additives on the fresh weight and root length of *D. moniliforme* seedlings following 4, 5, and 6 months of culture.

Medium	Organic Additive	Fresh Weight (mg)			Root Length(cm)		
		4 Months	5 Months	6 Months	4 Months	5 Months	6 Months
MS	-	2 ^g^	1 ^d^	1 ^h^	0 ^f^	0 ^e^	0 ^h^
	AH	1 ^g^	1 ^d^	1 ^h^	0 ^f^	0 ^e^	0 ^h^
	BH	2 ^fg^	12 ^cd^	29 ^fh^	0.29 ^cdef^	0.68 ^de^	1.08 ^efg^
	CW	4 ^defg^	4 ^d^	7 ^gh^	0.1 ^ef^	0.1 ^e^	0.26 ^fgh^
	ABC	3 ^efg^	10 ^d^	19 ^gh^	0.32 ^cdef^	0.56 ^de^	0.72 ^fgh^
Hyponex	-	20 ^a^	92 ^a^	83 ^cd^	0.9 ^ab^	2.7 ^a^	3.12 ^ab^
	AH	15 ^ab^	53 ^b^	152 ^b^	1 ^a^	1.98 ^ab^	2.72 ^bc^
	BH	7 ^cdefg^	15 ^cd^	16 ^gh^	0.6 ^bcd^	1.22 ^cd^	1.3 ^def^
	CW	18 ^a^	92 ^a^	201 ^a^	0.66 ^abc^	2.6 ^a^	4.0 ^a^
	ABC	9 ^bcde^	14 ^cd^	53 ^ef^	0.47 ^cde^	1.22 ^cd^	1.92 ^cde^
OMM	-	7 ^cdefg^	5 ^d^	4 ^gh^	0.1 ^ef^	0.14 ^e^	0.17 ^gh^
	AH	8 ^cdef^	11 ^d^	11 ^gh^	0.13 ^ef^	0.26 ^e^	0.3 ^fgh^
	BH	10 ^bcd^	17 ^cd^	107 ^c^	0.53 ^cd^	1.19 ^cd^	3.1 ^ab^
	CW	11 ^bc^	16 ^cd^	24 ^gh^	0.27 ^def^	0.22 ^e^	0.36 ^fgh^
	ABC	9 ^bcdef^	34 ^bc^	67 ^de^	0.42 ^cde^	1.42 ^bc^	2.26 ^bcd^
Two-way ANOVA	Medium	<0.001	<0.001	<0.001	<0.001	<0.001	<0.001
	Organic additive	<0.001	<0.001	<0.001	0.164	0.082	<0.001
	Medium organic additive	<0.001	<0.001	<0.001	<0.001	<0.001	<0.001

^1^ MS: Murashige and Skoog; Hy: Hyponex; OMM: Orchid Maintenance Medium; AH: apple homogenate; BH: banana homogenate; CW: coconut water; ABC: all three organic supplements. ^2^ Letters indicate the significant differences based on Tukey’s Honestly Significant Difference (HSD) test (*p*-value < 0.05). ^3^ *p*-values from a two-way analysis of variance (ANOVA) for all combinations of media and organic additives in each month.

## Data Availability

Data are contained within the article.
